# Dietary format alters fecal bacterial populations in the domestic cat (*Felis catus*)

**DOI:** 10.1002/mbo3.60

**Published:** 2013-01-07

**Authors:** Emma N Bermingham, Wayne Young, Sandra Kittelmann, Katherine R Kerr, Kelly S Swanson, Nicole C Roy, David G Thomas

**Affiliations:** 1 Food Nutrition & Health, Food & Bio-based Products Group, AgResearch GrasslandsPalmerston North, 4442, New Zealand; 2 Rumen Microbiology, Animal Nutrition & Health Group, AgResearch GrasslandsPalmerston North, 4442, New Zealand; 3 Division of Nutritional Sciences, University of Illinois, Urbana-ChampaignUrbana, Illinois, 61801, USA; 4 Department of Animal Sciences, University of Illinois, Urbana-ChampaignUrbana, Illinois, 61801, USA; 5 The Riddet Institute, Massey UniversityPalmerston North, 4442, New Zealand; 6 Centre of Feline Nutrition, Institute of Food, Nutrition and Human Health, Massey UniversityPalmerston North, 4442, New Zealand

**Keywords:** Feline, fecal bacterial community, high-throughput nucleotide sequencing

## Abstract

The effects of short-term (5-week) exposure to wet or dry diets on fecal bacterial populations in the cat were investigated. Sixteen mixed-sex, neutered, domestic short-haired cats (mean age = 6 years; mean bodyweight = 3.4 kg) were randomly allocated to wet or dry diets in a crossover design. Fecal bacterial DNA was isolated and bacterial 16S rRNA gene amplicons generated and analyzed by 454 Titanium pyrosequencing. Cats fed dry diets had higher abundances (*P* < 0.05) of Actinobacteria (16.5% vs. 0.1%) and lower abundances of Fusobacteria (0.3% vs. 23.1%) and Proteobacteria (0.4% vs. 1.1%) compared with cats fed the wet diet. Of the 46 genera identified, 30 were affected (*P* < 0.05) by diet, with higher abundances of *Lactobacillus* (31.8% vs. 0.1%), *Megasphaera* (23.0% vs. 0.0%), and *Olsenella* (16.4% vs. 0.0%), and lower abundances of *Bacteroides* (0.6% vs. 5.7%) and *Blautia* (0.3% vs. 2.3%) in cats fed the dry diet compared with cats fed the wet diet. These results demonstrate that short-term dietary exposure to diet leads to large shifts in fecal bacterial populations that have the potential to affect the ability of the cat to process macronutrients in the diet.

## Introduction

The domestic cat is an obligate carnivore, evolving on high-protein, low-carbohydrate (CHO) diets (Zoran 2002; Ritchie et al. 2010). Obesity levels in domestic cats are between 25% and 40% (Scarlett et al. 1994) and are increasing (German 2006). Intestinal microbiota have been implicated in the development of obesity in humans and rodent models (Ley et al. 2006; Turnbaugh et al. 2006, 2008, 2009). This may be due, in part, to shifts in the intestinal microbiota, which may result in alterations in energy metabolism (Turnbaugh et al. 2008). Therefore, there is increasing interest in the effects of diet on the intestinal microbiota profiles of the domestic cat, in order to ascertain any effects of metabolic disturbances.

Other than two studies performed recently (Barry et al. 2012; Hooda et al. 2012), most of the studies that have investigated the effects of diet composition on fecal microbiota in adult cats have focused on specific bacterial species using gel- or PCR-based methods. Several of these studies have evaluated the effects of prebiotics or dietary fiber (Barry et al. 2010; Kanakupt et al. 2011), while others have focused on protein : CHO ratios (Lubbs et al. 2009; Vester et al. 2009; Hooda et al. 2012). Lubbs et al. (2009), for example, reported that high-protein diets increased *Clostridium perfringens* and *Fusobacterium* and decreased *Bifidobacterium* populations in the feces of adult cats. In weaned kittens fed the same diets, high dietary protein decreased fecal *Escherichia coli*, *Bifidobacterium,* and *Lactobacillus* populations (Vester et al. 2009). Changes in dietary fiber also affect bacterial populations in the domestic cat, including decreased *Fusobacterium* species (Bueno et al. 2000). Pectin supplementation in adult cats increased the concentrations of *C. perfringens, E. coli,* and *Lactobacillus* spp., while fructooligosaccharide supplementation increased *Bifidobacterium* and decreased *E. coli* concentrations (Barry et al. 2012).

To our knowledge, all the studies investigating the effects of diet on bacterial composition utilizing next-generation sequencing in cats and dogs have examined the effects of dry diets (Barry et al. 2012; Hooda et al. 2012; Tun et al. 2012), with only preliminary investigations into the differences in bacterial composition between raw (meat) and kibbled diets fed to dogs reported (Beloshapka et al. 2011). Recent investigations focused on the composition and function of the intestinal microbiota of cats have shown differences at the phylum level between cats maintained in a research colony and cats living in a domestic setting (Tun et al. 2012). Typically, domestic cats are fed standard wet or dry diets that may greatly differ in moisture, CHO, protein, and fat content. Therefore, we conducted this study to understand the effects of two common conventional diet formats (i.e., wet or dry) on the bacterial composition in the gastrointestinal tract of cats. Because these diets contained many ingredient and nutrient differences, bacterial shifts may not be attributed to any one aspect of the diet, but the diets as a whole. Preliminary data using denaturing gradient gel electrophoresis and DNA sequencing indicated that cats that were changed from a wet to a dry diet showed an 86% change in the fecal microbiota composition, with major changes in Fusobacteriaceae and Comamonadaceae observed (Bermingham et al. 2011). The use of next-generation sequencing may provide a much more detailed view of the dietary effects on the bacterial communities of domestic cats, and allow the identification of minor changes that were not able to be identified on a taxonomic level by means of using denaturing gradient gel electrophoresis alone.

The hypothesis was that short-term dietary changes would alter the bacterial populations within the digestive tract of the domestic cat. The aim of this study was to investigate the effects of a short-term (5-week) dietary exposure to wet and dry diets on the fecal bacterial population of domestic cats using next-generation sequencing.

## Materials and Methods

### Animals and diets

The protocol for this study was approved by the Massey University Animal Ethics Committee (MUAEC # 09/103). All cats were housed at the Centre for Feline Nutrition (Massey University, Palmerston North, New Zealand) according to the Animal Welfare (Companion Cats) Code of Welfare (2007). Prior to the study, all cats were maintained on wet diets as part of standard feeding practices at the Centre for Feline Nutrition. In order to ensure that the cats were clinically and physiologically healthy prior to the study commencing, a complete blood count and thyroid assessment was carried out on each cat (data not shown).

Sixteen mixed-sex, neutered, domestic short-hair cats averaging 6 years of age (range = 1–10 years) and 3.4 kg bodyweight at the start of the study were used in a crossover design to determine the effects of short-term (5-week) exposure to wet (canned) or dry (kibbled) diets on the fecal bacterial communities. The cats were housed in two dietary treatments in adjacent colony cages (1400 × 2400 × 4400 cm). Cats were offered food ad libitum, receiving either a commercially available Association of American Feed Control Officials (AAFCO)-tested, wet diet or a dry diet (Table 1) for 5 weeks. At week 5, cats were placed in individual cages (80 × 80 × 110 cm) for 5 days to determine individual feed intake and fecal output. Fresh fecal samples were collected, mixed for homogeneity, subsampled, and stored at −85°C until bacterial community analysis. Cats were then changed to the alternative diet and the experimental procedure repeated.

**Table 1:** Macronutrient profile of commercially available Association of American Feed Control Officials (AAFCO)-tested maintenance diets fed to domestic short-hair cats (*Felis catus*)

Component	Dry diet^1^	Wet diet^2^
Dry matter (DM; % as is)	89.20	23.03
Crude protein (% DM)	32.91	41.87
Crude fat (% DM)	11.05	42.39
Ash (% DM)	8.28	8.81
Crude fiber (% DM)	1.88	1.62
NFE^3^ (% DM)	45.88	5.31
Gross energy (kcal/g DM)	4.80	6.66
Metabolizable energy^4^ (ME; kcal/g DM)	3.70	5.25

1 Ingredient list of dry diet (from pack): corn and corn protein; rice flour; meat products and meat derived from poultry, fish, lamb, and tuna; digest of poultry; chicken fat; palm stearine; dicalcium phosphate; salt; vitamins.

2 Ingredient list of wet diet (from pack): meat byproducts and meat derived from lamb, beef, chicken and mutton; vegetable protein; gelling agent; minerals; emulsifier; coloring; vitamins + taurine.

3 Nitrogen-free extract calculated by difference (100 − crude protein − crude fat − crude fiber − ash).

4 Determined using modified Atwater factors of crude protein (3.5 kcal ME/g DM), crude fat (8.5 kcal ME/g DM), NFE (3.5 kcal ME/g DM).

Apparent total intestinal digestibility of dietary energy and macronutrients (crude protein, crude fat, crude fiber, and ash; nitrogen-free extracts by difference) were determined for each diet. Individual food intake and refusals and fecal output were recorded daily. Total feces were collected over the 5-day collection period and frozen (−20°C), freeze dried, and ground to a fine powder using an electric grinder (Model CG-2; Breville, Oldham, UK), before analysis. Diet and fecal samples were analyzed for moisture using a convection oven at 105°C (AOAC 930.15, 925.10) and ash using a furnace at 550°C (AOAC 942.05). Crude protein and crude fat were determined using the Leco total combustion method (AOAC 968.06) and acid hydrolysis/Mojonnier extraction (AOAC 954.02), respectively. Gross energy (kJ/g) was determined using bomb calorimetry. Crude fiber was determined using the gravimetric method (AOAC 978.10) and nitrogen-free extracts by difference (Table 1).

Nutrient digestibility (Wichert et al. 2009) and metabolizable energy intake (NRC 2006) were determined. Briefly, the digestibility of macronutrients was determined using % digestibility = [(content in diet − content in feces)/content in diet] × 100. Metabolizable energy intake was calculated by correcting gross energy (determined via bomb calorimetry) content of the diet by energy digestibility and crude protein content (Bermingham et al. 2012).

### Bacterial community analysis of cat feces

Nucleic acids were extracted from feces (30 mg) with a combined bead-beating and phenol/chloroform protocol (Kittelmann and Janssen 2010). After bead-beating (1:1 sample/weight of zirconium beads) using a FastPrep FP120 (Qbiogene, Carlsbad, CA), cells were chemically disrupted with phenol/chloroform/isoamyl alcohol (25:24:1) and chloroform/isoamyl alcohol (24:1). DNA was precipitated from the aqueous phase with polyethylene glycol (30%). The DNA pellet was washed with 70% ice-cold ethanol, dried, and resuspended in 100 μL of molecular biology-grade water. Extracted DNA was quantified using a Nanodrop ND-1000 spectrophotometer (NanoDrop Technologies, Wilmington, DE).

### Bar-coded amplification of bacterial 16S rRNA genes and amplicon pooling

Primers Ba9F (5′-GAG TTT GAT CMT GGC TCA G-3′) (Weisburg et al. 1991) and Ba515Rmod1 (5′-CCG CGG CKG CTG GCA C-3′) modified from Lane et al. (1985) for PCR amplification of bacterial 16S rRNA genes were synthesized by Integrated DNA Technologies (Coralville, IA). Primers containing the Roche GS FLX adaptors A (5′-CCA TCT CAT CCC TGC GTG TCT CCG ACT CAG-3′) or B (5′-CCT ATC CCC TGT GTG CCT TGG CAG TCT CAG-3′) were used for Titanium sequencing (Rius et al. 2012). A two-base linker sequence between the bar code and the bacteria-specific primer, and a unique 12-base error-correcting bar code was attached to adaptor A for sample identification (Fierer et al. 2008). Each PCR reaction contained 40 μL of Taq PCR MasterMix (Qiagen, Hilden, Germany), 28 μL non-bar-coded primer (0.6 μmol/L), and 8 μL of bar coded primer (2 μmol/L). Before the addition of template DNA, a 19-μL aliquot was transferred into a sterile tube to serve as no-template negative control. The remaining 57 μL were spiked with 3 μL of DNA at a concentration between 20 and 40 ng/μL and divided into three aliquots of 20 μL. Amplification was performed as follows on a Mastercycler proS (Eppendorf, Hamburg, Germany): initial denaturation at 95°C for 2 min, 30 cycles of denaturing (95°C, 20 sec), annealing (52°C, 20 sec), and elongation (72°C, 1 min), and a final 7-min extension at 72°C. Triplicate PCR products were pooled, and correct sizes of PCR products and signal absence from the negative controls were verified by agarose gel electrophoresis. Subsequently, amplicons derived from the samples were purified using a High Pure PCR product purification kit (Roche Diagnostics, Mannheim, Germany), quantified using the Quant-iT dsDNA BR assay kit (Invitrogen, Carlsbad, CA) on a Qubit fluorometer (Invitrogen) and pooled in equimolar ratio into a single pool. The amplicon pool was sent to Macrogen (Seoul, Korea) for Titanium pyrosequencing on a 454 Life Sciences Genome Sequencer FLX machine (454 Life Sciences, Branford, CT).

### Bionumerics and statistics

Sequences were analyzed using the Quantitative Insights into Microbial Ecology (QIIME) version 1.3 pipeline using default parameters (Caporaso et al. 2010). Sequences passing quality control metrics were assigned to samples according to their 12-bp bar codes. Sequences sharing a minimum pair-wise similarity of 97% were binned into operational taxonomic units. Representative sequences from each operational taxonomic unit were aligned using PyNAST and a phylogenetic tree constructed using FastTree. Taxonomy for each operational taxonomic unit was assigned using the Ribosomal Database Project classifier with a support threshold of 80% (Wang et al. 2007). Beta diversity between samples was compared by Principal Coordinate analysis of weighted UniFrac distances. Bacterial abundance was tested using the Kruskal–Wallis rank sum test with diet as the main effect. Bodyweight was analyzed using REML variance components analysis (GenStat v12). Results are reported as mean and associated standard error of the mean (SEM) and were considered significant at *P* < 0.05 and a trend between *P* > 0.05 and *P* < 0.10.

## Results

### Metabolizable energy intake and nutrient digestibility

Metabolizable energy intake was different (*P* < 0.001) between diets (73.6 kcal/kg vs. 130.7 kcal/kg bodyweight/day in cats maintained on the dry and wet diets, respectively). The digestibility of crude fat and energy were not different between diets, whereas dry matter (DM) and crude protein digestibility were lower in cats fed dry diets (Table 2). Bodyweight was not significantly affected by diet (3.5 kg vs. 3.5 kg; SEM 0.12) in dry versus wet cats, respectively.

**Table 2:** Apparent total tract macronutrient digestibility (%) of the wet and dry diet fed to domestic short-hair cats (*Felis catus*)

	Dry (*n* = 16)	Wet (*n* = 16)	Pooled SEM	*P*-value
Dry matter	73.7	77.1	0.01	0.04
Energy	75.8	77.2	0.01	0.44
Protein	73.4	82.7	0.01	0.001
Fat	82.3	86.6	0.02	0.20

### Fecal bacterial profiles

**Figure 1:**
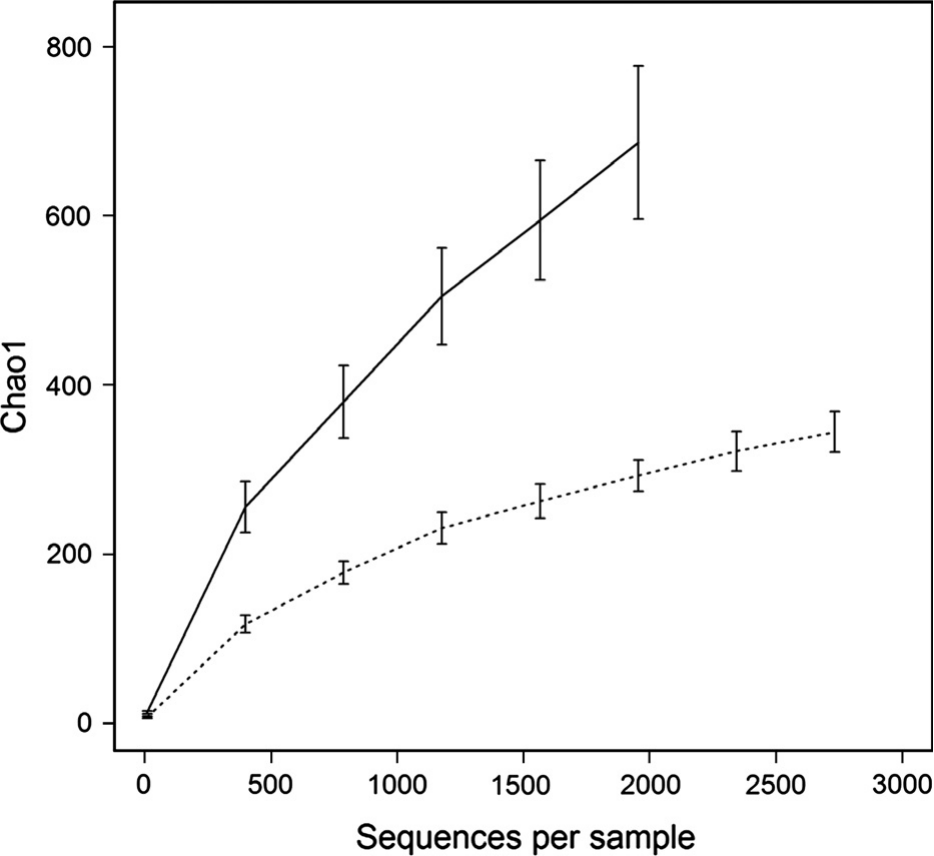
The effects of short-term exposure to a wet or dry diet on faecal microbial diversity. The rarefaction curve indicates the faecal microbiota CHAO1 diversity index (Chao1 index at 97% sequence identity cut-off) observed over the number of sequences sampled between cats fed wet (–) and dry (—) diets. Data are reported as means ± SEM (*n* = 16 cats per treatment).

Pyrosequencing of bacterial 16S rRNA gene amplicons resulted in a total of 147,703 sequences, with an average of 4616 (range = 2175–8223) sequences per sample. The number of operational taxonomic units identified was 3924. Rarefaction measures (CHAO) indicated that the diversity of the bacterial population in cats fed dry diets were lower compared with cats fed wet diets (Fig. 1).

The effects of diet format on fecal bacterial composition at the phylum level are shown in Table 3. The three most abundant phyla in cats fed the dry diet were Firmicutes, Actinobacteria, and Bacteriodetes, whereas in cats fed the wet diet, Firmicutes and Fusobacteria were the predominant phyla. Cats fed the dry diet had higher proportions of Actinobacteria (*P* < 0.05) and lower proportions of Fusobacteria, Proteobacteria, and unclassified bacteria (*P* < 0.05) compared with cats exposed to the wet diet.

**Table 3:** The effects of short-term exposure to a wet or dry diet on fecal bacterial phyla (% of total reads) in adult domestic short-hair cats *(Felis catus*). *P*-value indicates significance of Kruskal–Wallis rank sum test, and *q*-value indicates false discovery rate multiple-testing adjusted *P*-value

Taxon	Dry (*n* = 16)	Wet (*n* = 16)	Pooled SEM	*P*-value	*q-*value
Actinobacteria	16.5	0.1	3.8	0.011	0.068
Bacteroidetes	8.7	15.9	4.7	0.010	0.062
Firmicutes	73.6	57.6	7.7	0.090	0.539
Fusobacteria	0.3	23.1	2.9	<0.000	<0.000
Proteobacteria	0.4	1.1	0.2	0.008	0.047
Unclassified bacteria	0.5	2.1	0.2	<0.000	0.002

A total of 28 bacterial families were identified in this study. Of these, 17 were affected by diet format (data not shown). Major shifts associated with feeding dry diets included higher proportions (as a percentage of total reads) of Lactobacillaceae (32.13% vs. 0.15% [SEM 0.038] in the dry and wet cats, respectively), Veillonellaceae (23.93% vs. 2.26% [SEM 0.035] in the dry and wet cats, respectively), and Coriobacteriaceae (16.5% vs. 0.13% [SEM 0.038] in the dry and wet cats, respectively) populations. In cats fed the wet diet, major shifts were observed in abundances of Peptostreptococcaeae (3.02% vs. 30.24% [SEM 0.028]) in the dry and wet cats, respectively, and Fusobacteriaceae (0.29% vs. 23.10% [SEM 0.029]) in the dry and wet cats, respectively, with increases in the abundances of both these families.

Forty-six bacterial genera were identified in this study. Short-term exposure to dietary format significantly affected the proportions of 30 of these. There were 15 genera identified in cats fed the wet diet that were not present in the cats fed the dry diet. In cats fed the dry diet, the top five genera identified were *Lactobacillus*, *Megasphaera*, *Olsenella*, *Prevotella*, and *Streptococcus*. In the cats fed the wet diet, the predominant genera were *Peptostreptococcus*, *Fusobacterium*, *Clostridium*, and *Bacteroides*.

Of the classified genera affected, the dry diet increased the levels of *Lactobacillus*, *Megasphaera,* and *Olsenella*. Cats fed the wet diet had higher levels of *Blautia*, *Bacteroides, Faecalibacterium*, *Sutterella*, and *Sporacetigenium* (Table 4).

**Table 4:** The effects of short-term exposure to a wet or dry diet on fecal bacterial genera (% total reads) in adult domestic short-hair cats *(Felis catus*). *P*-value indicates significance of Kruskal–Wallis rank sum test, and *q*-value indicates false discovery rate multiple-testing adjusted *P*-value

Phyla/Family	Genera	Dry (*n* = 16)	Wet (*n* = 16)	Pooled SEM	*P*-value	*q*-value
Actinobacteria						
Coriobacteriaceae	*Olsenella*	16.4	0.0	3.8	0.002	0.105
Coriobacteriaceae	*Collinsella*	0.1	0.1	0.0	0.461	0.999
Bacteroidetes						
Prevotellaceae	*Prevotella*	7.5	4.5	2.8	0.228	0.999
Bacteroidaceae	*Bacteroides*	0.6	5.7	1.3	<0.000	0.003
Prevotellaceae	Unclassified Prevotellaceae	0.3	4.7	0.8	<0.000	0.001
Other	Unclassified Bacteroidales	0.2	0.7	0.2	0.308	0.999
Porphyromonadaceae	Odoribacter	0.1	0.1	0.0	0.006	0.293
Other	Unclassified Bacteroidetes	0.0	0.1	0.0	0.002	0.085
Porphyromonadaceae	Parabacteroides	0.0	0.1	0.0	0.002	0.105
Porphyromonadaceae	Unclassified Porphyromonadaceae	0.0	0.1	0.0	0.007	0.311
Firmicutes						
Lactobacillaceae	*Lactobacillus*	31.8	0.1	3.8	<0.000	<0.000
Veillonellaceae	*Megasphaera*	23.0	0.0	3.2	<0.000	<0.000
Streptococcaceae	*Streptococcus*	6.7	0.6	2.3	0.056	0.999
Peptostreptococcaceae	Unclassified Peptostreptococcaceae	2.7	28.9	2.8	<0.000	<0.000
Erysipelotrichaceae	Catenibacterium	2.7	0.3	0.8	0.062	0.999
Other	Unclassified Clostridiales	1.4	8.3	0.8	<0.000	<0.000
Other	Unclassified Firmicutes	1.0	0.2	0.1	<0.000	0.012
Lachnospiraceae	Unclassified Lachnospiraceae	0.6	2.9	0.3	<0.000	0.005
Veillonellaceae	*Megamonas*	0.6	0.8	0.3	0.073	0.999
Lactobacillaceae	Unclassified Lactobacillales	0.5	0.0	0.1	<0.000	<0.000
Clostridiaceae	*Clostridium*	0.5	6.1	2.4	<0.000	0.003
Lactobacillaceae	Unclassified Lactobacillaceae	0.3	0.0	0.0	<0.000	<0.000
Peptostreptococcaceae	*Sporacetigenium*	0.3	1.3	0.3	0.086	0.999
Veillonellaceae	Unclassified Veillonellaceae	0.3	1.2	0.2	0.001	0.027
Incertae Sedis XIV	*Blautia*	0.3	2.3	0.4	<0.000	0.002
Peptococcaceae	Unclassified Peptococcaceae	0.2	1.0	0.2	0.004	0.183
Erysipelotrichaceae	*Solobacterium*	0.2	0.1	0.0	0.008	0.367
Incertae Sedis XIII	*Mogibacterium*	0.1	0.0	0.0	0.070	0.999
Eubacteriaceae	*Eubacterium*	0.1	0.4	0.1	0.017	0.804
Other	Unclassified Bacilli	0.1	0.0	0.0	<0.000	0.005
Erysipelotrichaceae	Unclassified Erysipelotrichaceae	0.1	0.2	0.0	0.013	0.609
Veillonellaceae	*Allisonella*	0.0	0.2	0.1	0.288	0.999
Ruminococcaceae	*Faecalibacterium*	0.0	0.5	0.1	<0.000	0.001
Ruminococcaceae	Unclassified Ruminococcaceae	0.0	1.3	0.1	<0.000	<0.000
Ruminococcaceae	*Oscillibacter*	0.0	0.1	0.0	0.006	0.267
Clostridiaceae	Unclassified Clostridiaceae	0.0	0.1	0.0	0.001	0.068
Lachnospiraceae	*Dorea*	0.0	0.1	0.0	0.003	0.159
Enterococcaceae	*Enterococcus*	0.0	0.1	0.0	0.026	0.999
Lachnospiraceae	*Roseburia*	0.0	0.1	0.0	<0.000	0.003
Erysipelotrichaceae	*Allobaculum*	0.0	0.2	0.1	0.008	0.364
Fusobacteria						
Fusobacteriaceae	Unclassified Fusobacteriaceae	0.3	22.7	2.9	<0.000	<0.000
Fusobacteriaceae	*Fusobacterium*	0.0	0.4	0.1	0.000	0.010
Proteobacteria						
Succinivibrionaceae	*Anaerobiospirillum*	0.1	0.4	0.2	0.006	0.291
Enterobacteriaceae	*Escherichia/Shigella*	0.1	0.1	0.1	0.658	0.999
Alcaligenaceae	*Sutterella*	0.0	0.6	0.1	<0.000	0.001
Other						
Other	Unclassified bacteria	0.5	2.1	0.2	<0.000	0.014

**Figure 2:**
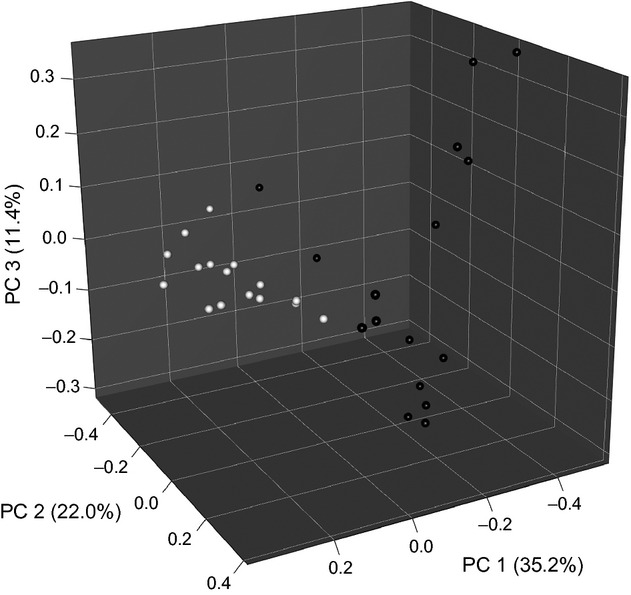
Principal Coordinate Analysis plot of weighted Unifrac phylogenetic distances showing the similarities between bacterial communities of cats fed dry (white) or wet (black) diets. Axes indicate percentage of variation explained by each principal coordinate.

Principal coordinate analysis of Unifrac distances showed that community profiles were clearly separated by diet (Fig. 2).

## Discussion

This is the first report that details the effects of dietary format on fecal bacterial populations in the domestic cat using next-generation sequencing. This study identified a dramatic shift in fecal bacterial communities induced by a 5-week exposure to a dry or wet diet with greater Actinobacteria and lower Fusobacteria and Proteobacteria observed in cats exposed to the dry diet. Five bacterial phyla were identified in the feces of cats, consistent with other studies investigating the intestinal microbiota in healthy cats (Ritchie et al. 2008, 2010; Desai et al. 2009; Handl et al. 2011). The phyla identified in cats fed the wet diet in this study were similar to those fed the dry diet, but the proportion of phyla changed between dietary formats. Tun et al. (2012) showed that in client-owned cats fed dry diets, Bacteroidetes (68%), Firmicutes (13%), and Proteobacteria (6%) were the predominant phyla, whereas data from laboratory cats (including this study) had higher Firmicutes (57–78%) and lower Bacteroidetes (0.2–16%) populations (Ritchie et al. 2010; Hooda et al. 2012). It is unknown whether this reflects a difference in the laboratory cats per se compared with client-owned cats, or whether differences are due to environmental differences in microbial exposure (e.g., grooming), diet, or methodological differences (e.g., primer design).

High protein : CHO diets (approximately 50% DM crude protein) decreased Actinobacteria and increased Fusobacteria levels in the growing kitten (Hooda et al. 2012), similar to the effects observed in the wet diet (high protein : CHO; 42:5% DM) in this study. While changes in phyla reported in Hooda et al. (2012) were similar to those observed in the adult cats fed wet diets in this study, differences were noted in bacterial genera between the two studies. For example, Hooda et al. (2012) observed increased *Dialister*, *Acidaminococcus*, *Bifidobacteria*, *Megasphaera,* and *Mitsuokella* with moderate protein : CHO diets (approximately 34% DM crude protein), which were not observed in this study. These differences may reflect differences in age (growing vs. adult cats in this study), diet (dry vs. wet format), or living conditions. It is possible that different bacterial species may have similar functions; however, functional data were not described for either Hooda et al. (2012) or this study, thereby emphasizing the increasing importance of understanding functional differences underlying the changes in bacterial composition.

A major genus of interest in the phylum Actinobacteria is the *Bifidobacterium*; species within this genus are thought to play a role in intestinal health. In contrast to some recent next-generation sequencing studies, bifidobacteria were not observed in either dietary group in this study. Bifidobacteria, which are carbolytic bacteria (Ritchie et al. 2010) capable of starch digestion (Suchodolski 2011), have been shown to be present in the intestine of the cat in other studies (Handl et al. 2011; Jia et al. 2011a; Hooda et al. 2012). It is possible that the absence of bifidobacteria observed in this study is due to primer bias (Palmer et al. 2007; Sim et al. 2012). In this study, the changes in the phylum Actinobacteria were mainly due to increased proportions of Coriobacteriaceae, namely the *Olsenella* genus, in cats fed the dry diet. Increases in Coriobacteriaceae abundance may have important consequences for health, as it has been associated with decreased blood glucose levels in mice (Claus et al. 2011) and increased blood non-high-density lipoprotein plasma concentrations and cholesterol absorption in hamsters (Martinez et al. 2009). High Coriobacteriaceae populations have also been observed in geriatric cats (8–14 years) fed dry diets (Jia et al. 2011b), although blood lipids and glucose were not measured in Jia et al. (2011a).

The levels of *Bacteriodetes* reported in the literature vary from very low levels (0.2%) in kittens fed moderate- or high-protein diets (Hooda et al. 2012) to 68% in client-owned cats (Tun et al. 2012). Although the overall abundance of the Bacteroidetes phylum remained unchanged between diets in this study, the composition within this phylum varied with diet on a lower taxonomic level (genus). For example, the large downward shift in the genus *Bacteroides* in cats fed the wet diet was largely compensated for by the upward shift in unclassified Prevotellaceae. This finding suggests that the *Bacteriodetes* phylum consists of diverse bacterial taxa capable of degrading both protein and CHO sources (Thomas et al. 2011). Further research with more in-depth sequencing is needed to identify the specific genera and species responding to diet and to identify any health implications it may have.

Within the Firmicutes phylum, differences between the *Clostridia* and *Bacilli* were observed, including members of the Lactobacillaceae (*Lactobacillus*), Peptostreptococcaceae, and Veillonellaceae (*Megasphaera*) families. In this study, fecal *Lactobacillus* populations were greater in cats fed the dry diet. *Lactobacillus* spp. are typically regarded as a beneficial group of microbes. Previous studies have reported higher fecal *Lactobacillus* in cats fed dry, moderate-protein, moderate-CHO diets compared with those fed high-protein, low-CHO diets (Hooda et al. 2012). In contrast, fecal *Lactobacillus* populations were lower in dogs fed raw meat versus dry, extruded diets (Beloshapka et al. 2011). These results from previous reports are in agreement with what was observed in cats of this study; this presumably reflects the saccharolytic nature of *Lactobacillus* and the CHO content of the dry diets used in these studies. In this study, *Clostridium* spp. increased in cats fed the wet diet, which concurs with previous studies that have shown elevated proportions of *Clostridium* in cats fed high-protein diets (Lubbs et al. 2009; Ritchie et al. 2010). These results suggest that members of the *Clostridium* genus in cats may be well adapted for proteolytic activity. Similarly, *Peptostreptococcus* spp., which have been associated with amino acid fermentation and breakdown (Suchodolski 2011), were also elevated in cats fed the wet diet. The large fecal bacterial shifts from CHO-utilizing to protein-utilizing bacteria observed in this study appear to reflect the differences in macronutrient profiles of the two diets. Proportions of *Megasphaera* (a member of the Veillonellaceae family), a major butyrate producer, were increased in cats fed the dry diet, which is in agreement with the data reported by Hooda et al. (2012), where cats fed a moderate-protein, moderate-CHO diet showed similar increases compared with those fed a high-protein, low-CHO diet.

Fusobacteria species contribute to amino acid fermentation and breakdown (Loesche and Gibbons 1968; Potrykus et al. 2008), which may explain the increased levels of this phylum in cats fed the wet diet in this study. Previous studies have observed low Fusobacteria populations, although diet was not detailed in these studies (Ritchie et al. 2010; Handl et al. 2011). However, higher Fusobacteria levels have been observed more recently by Hooda et al. (2012); however, primer design differed to that of this study. While Fusobacteria have been associated with a number of diseases in humans (Hooda et al. 2012), the cats in this study appeared to be healthy despite the high abundance of bacteria belonging to this phylum.

Changes in bacterial phylogeny observed in this study do not necessarily mean changes in function. Therefore, developing more robust data sets, including the analysis of functional changes (metagenomics and metatranscriptomics) is vital for understanding the effects of diet composition on the development of obesity phenotypes in domestic cats.
